# Anesthetic Approach for Non-Cardiac Procedures in Patients with a Left Ventricular Assist Device

**DOI:** 10.3390/jcm13185475

**Published:** 2024-09-15

**Authors:** Oscar Comino-Trinidad, Iria M. Baltar, Elena Sandoval, María Ángeles Castel, Marc Giménez-Milá

**Affiliations:** 1 Department of Anesthesiology, Critical Care and Pain Therapy, Hospital Clínic de Barcelona, 08036 Barcelona, Spain; imartinezb@clinic.cat (I.M.B.); magimene@clinic.cat (M.G.-M.); 2 Department of Cardiovascular Surgery, Hospital Clínic de Barcelona, 08036 Barcelona, Spain; esandova@clinic.cat; 3 Unit for Heart Failure and Heart Transplantation, Cardiovascular Institute, Hospital Clínic de Barcelona, 08036 Barcelona, Spain; macastel@clinic.cat; 4 Institut d’Investigacions Biomèdiques August Pi i Sunyer (IDIBAPS), 08036 Barcelona, Spain

**Keywords:** left ventricular assist device, advanced heart failure, non-cardiac surgery, sedation

## Abstract

**Background:** The use of durable left ventricular assist devices (LVADs) for advanced heart failure is increasing and a growing number of patients will require anesthesia for non-cardiac procedures (NCPs). The goal of this study was to describe our experience with NCPs for LVAD patients. **Methods:** All anesthetic procedures performed in LVAD patients at a single center were reviewed from 2014 to 2023. Perioperative management data and complications were assessed. **Results:** In total, 16 patients had an LVAD implanted and 9 (56.3%) patients underwent anesthesia for a total of 22 NCPs. Most of the procedures took place outside of the operating room, mainly in the endoscopy unit, as gastrointestinal endoscopy was the most common procedure (13, 59.2%). Sedation was provided in 17 procedures (77.3%). Standard monitoring was used in all cases, and invasive monitoring was applied just in cases of major surgeries. There were no intraoperative complications reported. Postoperative complications were recorded after eight (36.4%) of the procedures, consisting mainly of lower gastrointestinal bleeding after lower endoscopy, which increased the length of hospital stay. All procedures were performed by non-cardiac anesthesiologists. **Conclusions:** Our data suggest that, in most cases, adherence to standard anesthesia practices can be suitable for NCPs in LVAD patients.

## 1. Introduction

In Western countries, heart failure is still one of the leading causes of morbidity and mortality [1,2,3]. In the setting of limited organ supply or the presence of a contraindication for transplantation, durable left ventricular assist devices (LVADs) are the primary long-term mechanical circulatory support in patients with advanced cardiac failure [4]. LVADs might serve as a bridge to transplantation, a bridge to recovery or destination therapy [2,4,5]. The survival of LVAD recipients is constantly improving thanks to better device technology and safety [4,6]; thus, more patients are expected to be supported with LVADs and for longer periods of time. On the other hand, these patients require chronic anticoagulation therapy due to the possibility of thrombotic events [4,5,7]. This implies that, together with alterations inherent to the status of an LVAD carrier (acquired von Willebrand factor deficiency, impaired platelet aggregation and activation of fibrinolytic systems [5,7,8]), the risk of hemorrhagic complications is increased. For these reasons, a substantial number of LVAD patients will be diagnosed with conditions that may require non-cardiac procedures (NCPs) [5,9,10,11,12,13].

The unique physiology of patients with an LVAD, as well as the lack of evidence regarding the perioperative management of NCPs, can pose a challenge for anesthesiologists. The non-pulsatile flow might interfere with standard monitoring such as non-invasive blood pressure measurement and peripheral oxygen saturation measured by pulse oximetry. Moreover, the fine equilibrium between bleeding and thrombosis will imply relevant decisions as to when to stop anticoagulation and can have an impact on the development of intraoperative and postoperative complications. Also, they require specific attention with regard to hemodynamics, as the device has its own parameters regarding pulsatility, power and pump speed [5,14]. Safe and appropriate management of LVAD patients undergoing NCPs requires knowledge of the functioning of the device and careful planning.

Although some groups have published retrospective data on NCPs for LVAD patients [9,10,11,12,13], no reports have been published since 2017. The authors present their experience with the anesthetic and perioperative management of patients with an LVAD undergoing NCPs under the supervision of an anesthesiologist. Our goal is to describe the perioperative management of these patients, focusing on monitoring, anticoagulation and bleeding management, postoperative care and postoperative outcomes.

## 2. Materials and Methods

### 2.1. Study Design

A retrospective observational study was conducted at a single center. All patients carrying an LVAD since 2014 were retrospectively reviewed until December 2023. Data regarding anesthetic management for NCP after an LVAD implant were collected, including diagnostic and therapeutic endoscopies, all kinds of surgeries except for cardiac surgery and procedures outside of the operating room, as long as they were supervised by an anesthesiologist. For patients who received a heart transplant after an LVAD implant, we only collected data from procedures until the heart transplant.

### 2.2. Ethical Considerations

The study was conducted according to the guidelines of the Declaration of Helsinki, and approved by the Institutional Review Board of Hospital Clínic de Barcelona (protocol code HCB/2022/0012, approved on 18 February 2022). Patient consent was waived in accordance with the Institutional Review Board, as the majority of patients are not followed up with regularly by the Department of Anesthesiology and Reanimation in the center. Data were treated anonymously.

### 2.3. Data Collection

Demographic and preoperative data were collected, including age, sex, type of LVAD, the primary indication for the LVAD and current status (cardiac transplantation, dead or still carrying an LVAD). Outcomes included type of procedure, anesthesia technique and type of monitoring utilized, cardiac or non-cardiac anesthesiologist providing intraoperative care, admission to an intensive care unit (ICU) and time to discharge from the ICU, time to discharge from hospital, mortality during admission and mortality at 30 days after the procedure. Procedures were also classified as elective or emergent procedures. We also reviewed perioperative anticoagulation management, the use of vasopressors, blood and blood products transfusion, as well as perioperative complications such as bleeding, thrombosis or acute kidney failure. All data were obtained from electronic health records. As data were collected retrospectively, anesthesia was provided with no changes to clinical practice due to the study.

### 2.4. Statistical Analysis

Descriptive statistics are reported for all mentioned variables. Categorical variables are presented using frequency distributions and percentages, whereas continuous variables are shown as mean ± standard deviation and median and interquartile range when applicable. Statistical analysis was carried out using Stata version 15.

## 3. Results

### 3.1. Demographic Data

Between 2014 and 2023, 16 patients had an LVAD implanted at our institution and underwent a total of 22 NCPs. Demographic data are shown in Table 1. All patients were male, ranging from 20 to 75 years old at device implantation, with a mean age of 60 ± 13.2 years old. Two patients received a HeartMate II (HMII) (Abbott^®^, Pleasanton, CA, USA) in 2014 and 2016, and from 2017 onwards, all patients received a HeartMate 3 (HM3) (Abbott^®^, Pleasanton, CA, USA). The main indication for an LVAD implant was heart failure due to ischemic heart disease, followed by dilated cardiomyopathy to a lesser extent. One patient required an LVAD as a bridge to transplant due to complications of Fallot tetralogy, which corresponds to the youngest patient at 20 years old. Out of the nine (56.3%) patients who received an LVAD as a bridge to transplant, four (44.4%) received a heart transplant by the end of 2023, and none have died while on the waiting list for a heart transplant.

### 3.2. Non-cardiac Procedures and Type of Anesthesia

Out of the 16 patients in our case series, 9 (56.3%) had to undergo a total of 22 NCPs under the supervision of an anesthesiologist after LVAD implantation. The most frequent NCP was gastrointestinal endoscopy (GIE) (13 procedures, 59.2%). A breakdown of the type of procedure is shown in Table 2. Although the majority of patients underwent none or just one NCP (median of one NCP per patient [0;1]), there were three who received multiple NCPs: one underwent a total of four GIEs due to polypectomies; another one underwent a total of eight procedures consisting of multiple GIEs in the context of diagnosis and follow-up of a rectal tumor, as well as a resection and a posterior colostomy; the last one underwent a total of four procedures during LVAD implant admission due to thrombotic (endovascular cerebral procedures) and bleeding complications (tracheostomy bleeding).

Procedural characteristics are shown in Table 3. Most of the procedures (17, 77.3%) took place outside of the operating room, mainly in the endoscopy unit (13, 59.1%), but also in the interventional radiology unit (4, 18.2%). In total, 16 (72.7%) procedures were performed under sedation, which was the most frequent type of anesthesia, followed by general anesthesia (5, 22.7%). One case (4.5%) was performed with a subcostal transversal abdominal block and sedation (LVAD driveline debridement). A total of 13 out of the 16 (81.3%) procedures performed under sedation were carried out with propofol and remifentanil in target-controlled infusion (Schnider model for propofol, Minto model for remifentanil), with mean target concentrations of 1.5 ± 0.4 μg/mL and 1.2 ± 0.3 ng/mL, respectively. The maximum target concentration was 2.0 μg/mL for propofol and 1.6 ng/mL for remifentanil. Just 5 (55.6%) of the 9 patients who underwent NCPs carried an implantable cardioverter-defibrillator (ICD), which was deactivated in 4 (18.2%) of the 22 procedures.

Patients were admitted specifically for the procedure in 13 (59.1%) cases, 6 (27.3%) procedures were performed during admission for LVAD implantation and 3 (13.6%) during admission for other reasons. Regarding emergency, nine (40.1%) procedures were considered emergent, three of them related to thrombotic events (cerebral thrombectomy and arteriography) and six to bleeding events (cystoscopy, bronchial artery embolization, tracheostomy bleeding and lower GI bleeding).

### 3.3. Monitoring

**Figure 1:**
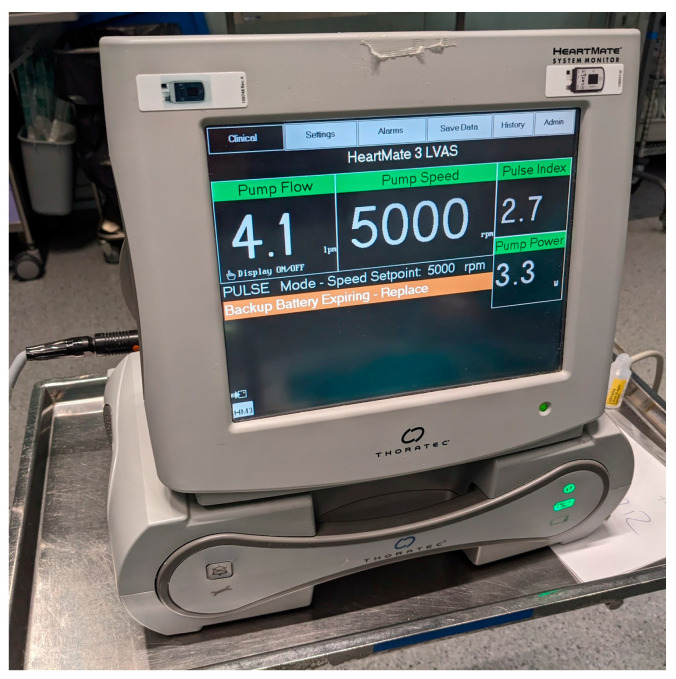
System monitor for HeartMate 3, showing hemodynamic parameters of pump performance (from left to right): pump flow (lpm), pump speed (rpm), pulse index, pump power (W).

Standard monitoring with pulse oximetry, a non-invasive blood pressure (NIBP) cuff (Philips^®^, Boeblingen, Germany and Dräger^®^, Lübeck, Germany), and an electrocardiogram were used in all cases. The bispectral index scale was used in four (18.2%) cases, all under general anesthesia. Capnography was used in all cases of general anesthesia, as well as in one GIE. Four (80%) out of the five cases performed in the operating room had LVAD parameter monitoring through a system monitor instead of the pocket system controller (Figure 1), but the monitor was not used at the endoscopy unit or the interventional radiology unit. Invasive arterial pressure monitoring with an arterial catheter was used in six procedures, although in one case, the patient already had the catheter placed because he was previously admitted to the ICU. All arterial cannulations were ultrasound-guided. A central venous line was specifically placed for the procedure in one (4.5%) case, but four (18.2%) patients already had one before the procedure. Complex monitoring, like transesophageal echocardiography (TEE) and regional cerebral oxygen saturation, was used in two (rectal neoplasia resection and colostomy) cases and one (rectal neoplasia resection) case, respectively. Anesthetic care was delivered in all cases by a non-cardiac anesthesiologist.

### 3.4. Hemostasis Management and Complications

Five (55.5%) out of the nine patients who underwent NCPs were under treatment with aspirin 100 mg daily, which was not interrupted in any procedure. Anticoagulation was initiated during admission after LVAD implantation. After hospital discharge, all patients were anticoagulated with acenocumarol.

When scheduled for an elective procedure, 7 (53.8%) out of 13 patients underwent bridge therapy with heparin, 3 (32.1%) discontinued acenocumarol with no bridge therapy and 3 (32.1%) did not stop it (diagnostic endoscopies with no therapeutic intervention planned). Bridge therapy with low-molecular heparin was administered at anticoagulant doses, either at a dose of 1 mg/kg every 12 h or 1.5 mg/kg every 24 h, and then stopped 24 h prior to the procedure. The indication for the procedure was bleeding in 2 (15.4%) out of the 13 elective procedures, which were both lower GIEs.

Regarding emergency procedures, in five (55.6%) out of nine patients’ acenocumarol was discontinued with no anticoagulation reversal (three lower endoscopies, cystoscopy and bronchial artery embolization). The remaining four procedures were carried out in a single patient, during LVAD implantation admission, who was under infusion of argatroban due to heparin-induced thrombocytopenia. Argatroban was not stopped for the procedures related to thrombosis (cerebral thrombectomy and arteriography), but was discontinued 4 h before the procedure related to bleeding (tracheostomy bleeding), and resumed 1 day afterward.

### 3.5. Intraoperative Complications

There were no intraoperative complications recorded, including no low-flow events. Blood transfusion was needed intraoperatively in three (13.6%) procedures. In these cases, the indication for surgery was bleeding (cystoscopy, bronchial artery embolization and tracheostomy bleeding). No other blood products were administered intraoperatively. Vasopressors (noradrenaline) were administered during five (22.7%) procedures, although just one patient required them postoperatively and could be withdrawn in less than 24 h.

### 3.6. Postoperative Period

Postoperative complications were recorded after eight (36.4%) of the procedures. The majority of postoperative complications consisted of lower GI bleeding and took place after elective lower endoscopies. There was a need for blood transfusion postoperatively in two (9.1%) cases. In one of them, bleeding was associated with acute kidney injury and superior mesenteric artery embolization was required, as well as plasma and platelets transfusion. There was one postoperative complication related to thrombosis (ischemic stroke), requiring a thrombectomy. One (4.5%) patient required continuation of vasopressor in the postoperative period and was admitted to the ICU (rectal neoplasia resection).

Postoperative care took place mainly in the conventional ward for 13 procedures (59.1%). In two (9.1%) procedures, patients were cared for in the ICU (rectal neoplasia resection and colostomy) and in five (22.7) procedures, patients were previously admitted to the ICU. Two (9.1%) patients had a diagnostic lower endoscopy on an outpatient basis, with no therapeutic intervention. No patients died during admission for the procedure or within 30 days after the procedure.

### 3.7. Gastrointestinal Endoscopies

GIEs were the most frequent NCP in patients carrying an LVAD. In total, 5 (31.3%) patients underwent a total of 13 GIEs (Table 4). The median duration was 29.2 ± 20.8 min, ranging from 10 (colonoscopy) to 75 min (colonoscopy for rectal neoplasia study with ultrasound and biopsy). The most frequent indication was GI bleeding. Other indications were the evaluation of a suspected neoplasia and a pre-transplant study. In nine (69.2%) GIEs, an invasive procedure was carried out (polypectomies, biopsies and treatment of angiodysplasias). In these cases, patients were admitted 2 to 5 days prior for anticoagulation bridge therapy. When no invasive procedure was planned, patients were admitted on an outpatient pathway with no change in anticoagulation therapy (two procedures).

All procedures were performed under sedation with spontaneous ventilation with propofol and remifentanil in target-controlled infusion. Standard monitoring (pulse oximetry, NIBP and electrocardiogram) was used. Capnography was used in one (7.7%) procedure. All patients received oxygen through the nasal cannula. No patients required vasoactive support or advanced airway management. Of the 9 GIEs with invasive procedures, 6 (66.7%) presented postprocedural GI bleeding as a complication, requiring longer hospital stay (up to 17 days) and a new GIE in 2 cases and a superior mesenteric artery embolization in 1 case. Two (33.3%) required postprocedural transfusion. When there was no bleeding as a postoperative complication, patients were discharged in 3 days. Thrombotic complications were not observed in any of the patients. As stated previously, none of the procedures were carried out by cardiac anesthesiologists.

## 4. Discussion

Although there have been previous reports of the perioperative management of patients with an LVAD undergoing NCPs [9,10,11,12,13,15], there have been none in recent years. Our study shows a single institutional experience of patients with an LVAD undergoing NCPs under the supervision of an anesthesiologist.

In our series, a majority of patients were equipped with HM3 devices, in contrast with previous reports, where HMII and HeartWare HVAD (Medtronic, Dublin, Ireland) were the most frequent devices and there were no or very few HM3s [10,13]. HMII is a continuous, axial-flow LVAD that used to be the most frequently implanted device. HM3 is a novel continuous-flow LVAD that uses a centrifugal flow and has a novel feature consisting of an artificial pulse generated by a designed algorithm, producing periodic variations in impeller speed and causing some degree of pulsatility, thereby reducing stasis and the potential for thrombus formation [5,14]. HeartWare HVAD is also a centrifugal pump device, but its distribution was ceased as a result of safety concerns associated with potentially fatal pump malfunctions from electrical faults causing battery failure [16].

Out of the 16 patients in our case series, just 9 (56.3%) underwent a total of 22 NCPs under the supervision of an anesthesiology. Most LVADs in our center have been implanted in recent years, which could explain why the majority were HM3 and almost half of them did not yet require NCPs. The most frequent procedure in our series was GIE, concurring with previous reports [10,11,12,13]. GI bleeding is very frequent among these patients due to different factors [4,8,17,18,19], and previous reports suggest that up to 30% of patients might present with GI bleeding [8].

Before the procedure, a complete preoperative assessment was carried out, as recommended [4,13,14]. A multidisciplinary approach including cardiologists, cardiac surgeons and anesthesiologists is crucial, and patients should have a recent check-up with the cardiac failure specialist, including LVAD-specific medical history, device interrogation and a recent cardiac ultrasound with a special focus on right ventricle function, inflow cannula position, to avoid suction events, and aortic valve opening [4,13,17]. A cardiac surgeon or a heart failure specialist should be informed and available for consultation during the procedure if a problem arises regarding the functioning of the LVAD [5,14]. Careful consideration should be taken when assessing bridge therapy to find the right equilibrium between bleeding and thrombosis risk [8,17,20].

Sedation with propofol and remifentanil in target-controlled infusion was the most frequent type of anesthesia performed mainly because GIE was the most frequent procedure. Titration of sedation is especially important in these patients to avoid hypotension. Sedation depth was measured according to patient response and breathing pattern as standard practice. As our sample suggests, a low target concentration might suffice in most cases, as the maximum target concentration was 2.0 μg/mL for propofol and 1.6 ng/mL for remifentanil.

Overall, general anesthesia with a secured airway was reserved for the few procedures that took place in the operation room. Interestingly, there was a case of driveline debridement that was carried out with a transversus abdominal plane block and sedation. The driveline is the connection between the pump and the external batteries and goes through the skin, and, as non-biological implantable material, it can become infected and require debridement. Driveline debridement is a common procedure in these patients, as reported in previous series [9,13], and a regional block might be an option to avoid hemodynamic changes related to general anesthesia in these cases. Regional and neuraxial anesthesia are not common strategies in these patients, as a frequent indication for a procedure is bleeding, which may impair coagulation and hemodynamics. In fact, there were no cases of neuraxial anesthesia reported in our series. Interestingly, there have been reports of neuraxial anesthesia for elective procedures in patients for whom anticoagulation bridging has been feasible, with no postoperative complications [21,22], and even in the emergency setting [13].

**Figure 2:**
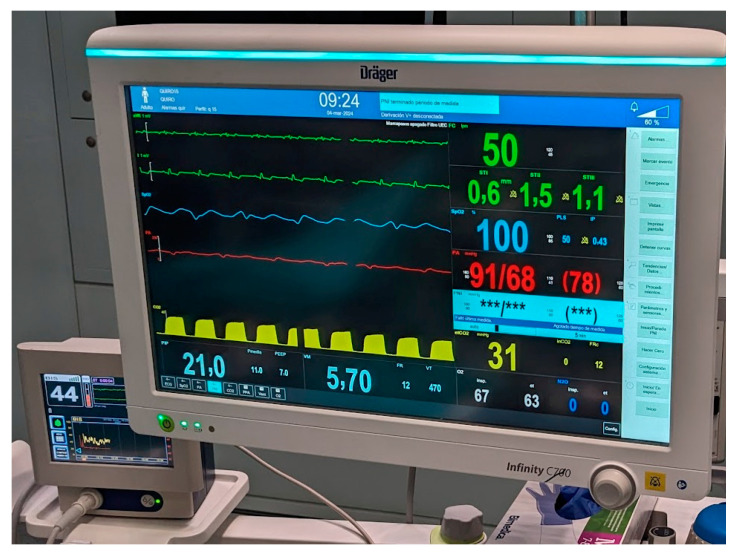
Intraoperative monitoring for non-cardiac surgery in a patient with a left ventricular assist device. Monitoring includes electrocardiogram (green), invasive arterial blood pressure (red) and pulse oximetry (blue). Pulsatility can be seen in both the pulse oximetry and the invasive blood pressure, although just half of the heartbeats produce a pulse.

Standard monitoring with pulse oximetry, NIBP and electrocardiogram was carried out in all cases, as recommended for any procedure. Capnography was also used in cases of general anesthesia and in the interventional radiology unit. The depth of anesthesia was measured with a bispectral index scale in four out of five cases of general anesthesia, and we recommend its use in all cases of general anesthesia. Arterial catheters and central venous lines were less frequently used. Overall, approximately a fourth of our patients had an arterial catheter during the procedure (Figure 2), which is less than reported in previous series [10,11,13], when it was used even in some cases of GIE.

NIBP has been shown to be accurate when obtained, even when just displaying the mean blood pressure [4,5,14], although it might not be appropriate when frequent shifts are anticipated. Patients should be asked if NIBP works on them, and it should be used prior to the start of the procedure to ensure that blood pressure can be obtained with this method. Arterial blood pressure control is paramount in these patients, as high mean arterial pressure (over 90 mmHg) can favor thrombotic events and even inverse the pump flow, having a devastating effect [5,14]. Arterial catheters should probably be used in major procedures and in patients for whom NIBP does not deliver a value of blood pressure, such as, for example, in patients with low pulsatility due absence of aortic valve opening [5,14,17]. Due to the lack of pulsatility, ultrasound-guided catheterization is often required. As shown by Stone et al. [11], the tendency over time is to use less invasive monitoring, as these patients are becoming more frequent and we are more familiarized with their hemodynamics.

TEE can be used as an intraoperative guidance, especially to help identify pump malfunction, assess left ventricular decompression, right ventricular dysfunction, suction events or guide fluid and inotropic therapy [5,17], although it is rarely used [11,13]. In our case series, it was used from the beginning on two occasions, during rectal neoplasia resection and colostomy. In the former case, the patient was required to be in a prone position. There have been reports of LVAD patients undergoing NCPs in the prone position [23,24], in which TEE was not only useful in guiding fluid therapy, but also helped to identify right ventricle outflow tract compression by LVAD outflow cannula. In our case, the prone position was well tolerated and just a mild hypotension requiring low-dose vasopressors was reported. Regional cerebral oxygen saturation was also used in this case, and although its use has not been extensively studied in LVAD patients, it might be useful to guide optimal blood pressure and as a parameter to control blood loss [17].

Key hemodynamic parameters of LVAD include pump flow (Lmin), pump speed (revolutions per minute), pulsatility index and pump power. Pump speed is fixed by the referring physician and it is determined according to the cardiac output needed by the patient without causing wall suction events. Pump power is the energy to maintain the speed and can indicate an increase in pump resistance to outflow. Pump flow is an estimate of cardiac output according to the pump power at a fixed pump speed. Finally, the pulsatility index is a dimensionless index used to measure the extent of pulsatility in the LVAD flow waveform, and it is affected directly by left ventricle preload and contractility, as well as heart rate and rhythm. These parameters help monitor pump function and can help diagnose systemic perfusion.

In the event of a hypotensive event, some authors have designed algorithms for its management, like the one designed by Feldman et al. [25] and adapted by Dalia et al. [5]. In the event of hypotension, vasodilation should be considered if a high flow is observed, reducing vasodilating drugs, using vasopressors to attain the desired blood pressure or treating the possible cause (such as sepsis). In the case of hypotension with low flow, hypovolemia should be the main concern, administering bolus fluids or blood if low hematocrit is present.

The electric scalpel does not affect the functioning of the LVAD. Caution should only be exercised in patients with an ICD, which should be disconnected in cases where an electric scalpel is required. Patients with an ICD in our series had the device therapy discontinued during the procedure and external defibrillator pads were placed, although they were not necessary in any of the cases.

Regarding GIE, all procedures were performed under sedation with an oxygen nasal cannula and standard monitoring. In previously published series, there have been cases of GIE performed under general anesthesia, some of them with an arterial catheter [11,12,13], or under the supervision of an anesthesiologist with a cardiac anesthesia background [26]. None of our patients presented any intraoperative complications and postoperative complications were related to bleeding. This could indicate that sedation under standard monitoring is safe and may suffice in this scenario, although the number of procedures performed is low compared to the previous series. There were even two cases in which no therapeutic or diagnostic intervention was planned or performed during the GIE (and thus, there was no change in anticoagulation), and the patients were treated on an outpatient basis. Postoperative GI bleeding after GIE was frequent when invasive procedures such as biopsies and polypectomies were performed, and some patients required a second procedure or even a packed red blood cell transfusion. In this scenario, the length of stay was considerably prolonged, suggesting that patients requiring invasive procedures should be closely monitored for the possibility of bleeding and may require a certain length of stay.

Transfusion requirements vary among series [9,10,11,13], but overall, packed blood cell transfusion was the most frequently transfused product in NCPs in patients with LVAD, ranging from 12 to 41% of the procedures. This is in concordance with bleeding being the most frequent indication for NCPs, as well as the most frequent postoperative complication. In our series, three (13.6%) patients required intraoperative blood transfusion (bleeding was the indication for the procedure) and two (9.1%) required it in the postoperative period (GIE in both cases). There was no transfusion of other blood products in contrast with the previous series [10,11,13].

On the other hand, although there have been reports of pump thrombosis and embolic stroke [10,19], complications related to thrombosis are rarely seen compared to bleeding. This might be due to different reasons, including the tendency of these patients to bleed as stated above, and also a more intensive anticoagulation management, as a pump thrombosis can be devastating [4,5,17]. Thrombotic risk seems to be related to low pump speed, specific surgical implant techniques and the absence of heparin bridging [17,19] and appears to be less frequent with HM3 devices compared to HMII [17]. In our series, there was a patient carrying an HM3 who presented with an embolic stroke requiring cerebral artery thrombectomy and posterior angiography, but otherwise, we had no other thrombotic complications.

Even though no intraoperative complications were observed, there was an important incidence of postoperative bleeding, although we did not observe in-hospital mortality or 30-day mortality after any procedure, which could indicate that bleeding was not severe or rightly handled. Postoperative care took place mainly in the conventional ward, and ICU admission was reserved for major procedures and patients admitted previously due to other conditions, mainly during initial admission for LVAD implantation.

The need for a specific cardiac anesthesia background to care for LVAD patients in NCPs has been thoroughly discussed previously in the literature. Initially, it was mainly cardiac anesthesiologists who cared for these patients, even for NCPs, although this has changed over time [10,11,26]. In a survey carried out by Sheu et al. [26], anesthesiologists state that education regarding LVAD patients is scarce, although it is more and more frequent to find these patients in the endoscopy unit and the operation room. In fact, in our series, all patients were cared for by non-cardiac anesthesiologists, although there was a cardiac surgeon available for major procedures and cardiac anesthesiologists were available for consultation. The most recent recommendations [11,14,17,19] suggest that non-cardiac anesthesiologists might be suitable to care for these patients in specialized centers where LVAD patients are frequent and support from cardiac surgeons, cardiac anesthesiologists and cardiac failure teams is available. Also, Stone et al. [11] suggest that regular training for non-cardiac anesthesiologists should take place in referral centers and that in cases with significant comorbidities, major procedures or when pharmacological support is required, a cardiac anesthesiologist should be available.

Our study is not without limitations. This is a single-center retrospective study with a limited number of individuals and procedures; therefore, it might not be generalizable. LVAD-specific parameters were not documented. In addition, the most used device in our center was HM3.

Despite our limitations, our cohort of NCPs in LVAD patients presents an updated overview of the perioperative period, with a special representation of the new HM3 device, which has not been as present in previously published series.

## 5. Conclusions

LVAD patients presenting for NCPs present certain peculiarities that should be taken into consideration when performing anesthetic procedures. As the number of these patients and their survival increases, these patients will be found more frequently in our everyday practice, especially in the endoscopy unit, as GI bleeding is very frequent in these patients. Sedation seems to be appropriate in this setting, as it was the most frequent anesthesia technique, and no intraoperative complications were observed. Special care should be taken regarding anticoagulation management, as bleeding is frequent both as an indication and as a postoperative complication, and thrombotic events can be devastating. In most cases, adherence to standard anesthesia principles and practices can be suitable and safe.

## Figures and Tables

**Table 1:** Patient demographics and baseline characteristics.

Demographic Data	Variable	*n* (%) of Patients ^1^
Total patients		16
Mean age (years)		60.2 ± 13.2
Sex	Male	16 (100.0)
	Female	0 (0.0)
LVAD type	HMII	2 (12.5)
	HM3	14 (87.5)
Indication of LVAD	Ischemic heart disease	11 (68.8)
	Dilated cardiomyopathy	4 (25.0)
	Fallot tetralogy	1 (6.3)
Goal of LVAD	Bridge to transplantation	9 (56.3)
	Destination therapy	7 (43.7)
Patients with implantable cardioverter-defibrillator		9 (56.3)

HMII: HeartMate II, HM3: HeartMate 3, LVAD: left ventricular assist device. ^1^ Except median age, which is shown as mean of years ± standard deviation.

**Table 2:** Non-cardiac procedures in LVAD patients.

Type of Procedure	*n* (%) of Procedures
Total procedures	22
Gastrointestinal endoscopy	13 (59.1)
Esophagogastroduodenoscopy ^1^	3 (13.6)
Colonoscopy ^1^	10 (45.5)
LVAD driveline debridement	1 (4.5)
Rectal neoplasia resection	1 (4.5)
Colostomy	1 (4.5)
Cerebral thrombectomy	2 (4.5)
Cerebral arteriography	1 (9.1)
Cystoscopy	1 (4.5)
Bronchial artery embolization	1 (4.5)
Tracheostomy bleeding	1 (4.5)

LVAD: left ventricular assist device. ^1^ Included in gastrointestinal endoscopy.

**Table 3:** Characteristics of non-cardiac procedures in LVAD patients.

Procedural Characteristics	*n* (%) of Procedures
Type of anesthesia	
Sedation	16 (72.7)
General anesthesia with endotracheal tube	3 (13.6)
General anesthesia with laryngeal mask	1 (4.5)
General anesthesia with tracheostomy	1 (4.5)
Regional block + sedation	1 (4.5)
Location of the procedure	
Endoscopy unit	13 (59.1)
Operating room	5 (22.7)
Interventional radiology unit	4 (18.2)
Monitoring	
Standard	22 (100.0)
Capnography	10 (45.5)
Bispectral index scale	4 (18.2)
Arterial catheter	6 (27.3)
Central venous line	5 (22.7)
Transesophageal ultrasound	2 (9.1)
Regional cerebral oxygen saturation	1 (4.5)
Care provided by cardiac anesthesiologist	
Yes	0 (0.0)
No	22 (100.0)
Urgency of procedure	
Elective	13 (59.1)
Emergent	9 (40.9)
Procedure during LVAD implantation admission	
Yes	6 (27.3)
No	16 (72.7)
Number of patients with complications	
Intraoperative	0 (0.0)
Postoperative	8 (36.4)
Complications	
Gastrointestinal bleeding	6 (27.3)
Ischemic stroke	1 (4.5)
Postoperative vasopressor dependence	1 (4.5)
Acute kidney injury	1 (4.5)
Blood transfusion	
Intraoperative	3 (13.6)
Postoperative	2 (9.1)
Use of vasopressors	5 (22.7)
Anticoagulation management	
Bridge therapy with heparin	7 (31.8)
Argatroban infusion	4 (18.2)
No bridge therapy	11 (50.0)
Postoperative care	
Ward	13 (59.1)
During ICU admission	5 (22.7)
ICU	2 (9.1)
Outpatient	2 (9.1)
Mortality during admission for the procedure	0 (0.0)
Mortality at 30 days after the procedure	0 (0.0)

ICU: intensive care unit. LVAD: left ventricular assist device.

**Table 4:** Gastrointestinal endoscopy characteristics in LVAD patients.

Procedural Characteristics	*n* (%) of Procedures
Total gastrointestinal endoscopies	13
Colonoscopy	10 (45.5)
Esophagogastroduodenoscopy	3 (13.6)
Total of patients undergoing endoscopic procedures	5 (23.3)
Indication ^1^	
Gastrointestinal bleeding	6 (46.2)
Evaluation of a suspected neoplasia	4 (30.8)
Pre-transplant study	2 (15.4)
Follow-up	1 (7.7)
Invasive procedure	9 (69.2)
Length of stay	
≤3 days	7 (53.8)
4–17 days	6 (46.2)
Monitoring	
Standard	13 (100.0)
Capnography	1 (7.7)
Complications	
Gastrointestinal bleeding	6 (46.2)
Other	0 (0.0)
Blood transfusion	
Postoperative	2 (15.4)
Intraoperative	0 (0.0)

LVAD: left ventricular assist device. ^1^ All variables of this table are measured by the number and the percentage of endoscopy procedures except the indication, which we preferred to express by the number and the percentage of patients.

## Data Availability

The raw data supporting the conclusions of this article will be made available by the authors on request.
